# The Role of Acyl-Glucose in Anthocyanin Modifications

**DOI:** 10.3390/molecules191118747

**Published:** 2014-11-14

**Authors:** Nobuhiro Sasaki, Yuzo Nishizaki, Yoshihiro Ozeki, Taira Miyahara

**Affiliations:** 1 Iwate Biotechnology Research Center, 22-174-4, Narita, Kitakami, Iwate 024-0003, Japan; E-Mail:n-sasaki@ibrc.or.jp; 2 Department of Biotechnology and Life Science, Tokyo University of Agriculture and Technology, 2-24-16 Nakacho, Koganei, Tokyo 184-8588, Japan; E-Mails: ynishizak@nihs.go.jp (Y.N.); miyahara@cc.tuat.ac.jp (T.M.)

**Keywords:** acyltransferase, anthocyanin, *Arabidopsis*, carnation, delphinium, glucosyltransferase

## Abstract

Higher plants can produce a wide variety of anthocyanin molecules through modification of the six common anthocyanin aglycons that they present. Thus, hydrophilic anthocyanin molecules can be formed and stabilized by glycosylation and acylation. Two types of glycosyltransferase (GT) and acyltransferase (AT) have been identified, namely cytoplasmic GT and AT and vacuolar GT and AT. Cytoplasmic GT and AT utilize UDP-sugar and acyl-CoA as donor molecules, respectively, whereas both vacuolar GT and AT use acyl-glucoses as donor molecules. In carnation plants, vacuolar GT uses aromatic acyl-glucoses as the glucose donor* in vivo*; independently, vacuolar AT uses malylglucose, an aliphatic acyl-glucose, as the acyl-donor. In delphinium and *Arabidopsis*, *p*-hydroxybenzoylglucose and sinapoylglucose are used* in vivo* as bi-functional donor molecules by vacuolar GT and AT, respectively. The evolution of these enzymes has allowed delphinium and *Arabidopsis* to utilize unique donor molecules for production of highly modified anthocyanins.

## 1. Introduction

Angiosperms display a wide range of flower colors and many species are exploited for horticultural purposes because of this characteristic. Flower colors commonly depend on three major plant pigments, namely, flavonoids/anthocyanins, betalains and carotenoids [1]. Of these, anthocyanins are responsible for the widest array of color varieties. The fundamental color of anthocyanins depends on the aglycon type. The six common anthocyanin aglycons are produced in angiosperms that have different pattern of the hydroxylation and methylation on B-ring [1]. Although angiosperms have only six anthocyanin aglycons (the basic skeletal form of anthocyanins), their flowers show considerable variability in color. Anthocyanins are subjected to diverse types of modifications, such as the position within the molecule of the modifying moiety, the type of attached sugar or organic acid, or a combination of these variable factors; through this diversity of possible modifications, a large range of differently colored anthocyanins can be generated. Furthermore, these modifications play important roles in the chemical stabilization of anthocyanin molecules in the vacuolar sap and in determining the color of the flowers in response to variations in vacuolar sap pH or to interactions with metal ions or aromatic organic acids in the vacuole [2]. The understanding of the reaction mechanisms to generate those complex anthocyanin molecules would help us to know how the plants acquire them in their evolutional process and to clarify the other metabolite biosynthetic pathways. The DNA manipulation technique based on that information would be useful to create a flower crops presenting the new color.

**Figure 1:**
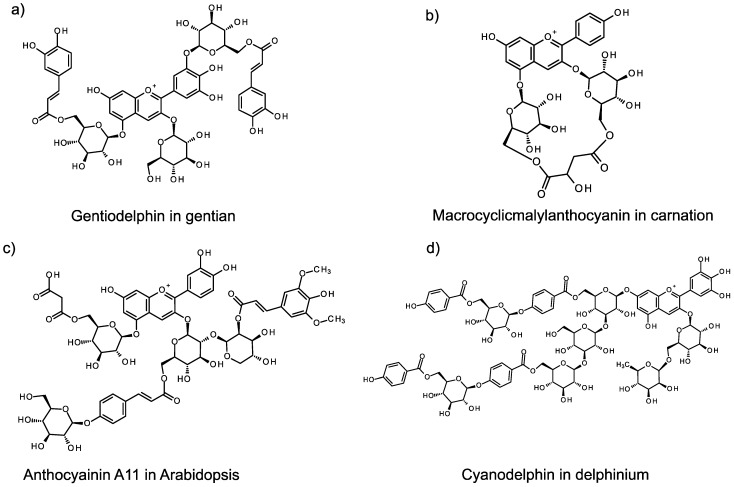
Representative anthocyanins showing modification by sugar and acyl groups.

Some plant species can produce complicated molecular anthocyanin structures, termed polyacylated anthocyanins, by attachment of multiple sugar and organic acid moieties (see Figure 1). As each step of anthocyanin modification is mediated by specific enzymes, the diversity of anthocyanin molecular structures is believed to have resulted from divergent evolution of those enzymes. In recent years, most of the enzymes, and the genes encoding them, for each reaction step to generate anthocyanin aglycons have been identified, and, currently, attempts to change and modulate flower colors using the information on gene sequences have been initiated [3].

Glycosylation of anthocyanidin and anthocyanin is largely catalyzed by family 1 glycosyltransferases (UGTs) that utilize UDP-sugars as the sugar donor and that are active in the cytosol [4]. To date, UGTs have been identified in many plant species [4]. A second modification system, acylation, is commonly catalyzed in the cytosol by anthocyanin acyltransferase (AT) that utilizes acyl-CoA as the acyl-donor. This type of acyltransferase is classified as a BAHD family protein, which characteristically displays benzoylalcohol acetyl transferase, anthocyanin-*O*-hydroxycinnamoyltransferase, anthoranilate-*N*-hydroxybenzoyl/benzoyltransferase and deacetyl-vindoline acetyltransferase activities [5]. The final modified structures of some anthocyanins, such as the polyacylated anthocyanin gentiodelphin of Japanese gentian (Figure 1), are accomplished through the activities of UGTs and an AT. All of the reaction steps necessary to synthesize gentiodelphin have now been identified [6]. In gentian flower petals, the synthesized anthocyanin aglycon is glucosylated at the 3-position and the 5-position. Two pathways have been identified: acylation of the glucose moiety at the 5-position in advance of 3'-glucosylation; and, 3'-glucosylation before the acylation of the glucose moiety at the 5-position [7]. Interestingly, acylation of both glucoses at the 5- and 3'-positions is catalyzed by the same enzyme [8,9,10]. Hence, gentiodelphin formation is achieved through modification of the aglycon by UGTs and a BAHD type AT (BAHD-AT).

**Figure 2:**
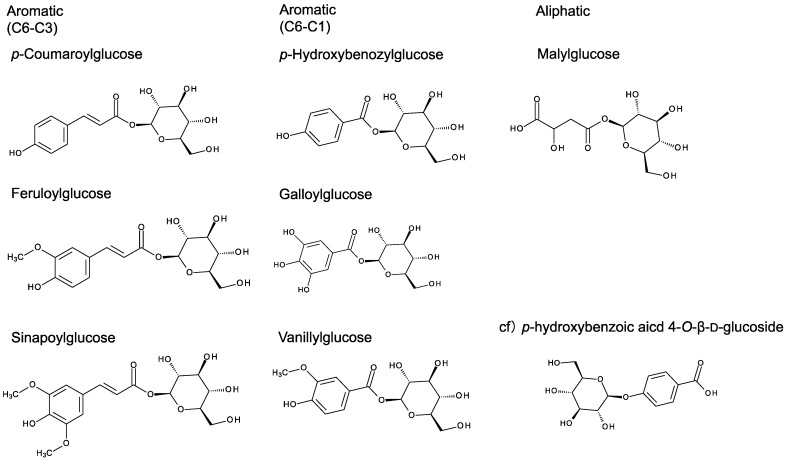
Representative chemical structures of acyl-glucoses.

Anthocyanin acylation has also been reported to be mediated by an enzyme that is clearly distinct from BAHD-AT. Carrot cells in suspension cultures produce the anthocyanin cyanidin 3-*O*-(6''-*O*-(6'''-*O*-sianpoyl-glucosyl)-2''-*O*-xylosyl)-galactoside (Cya 3-(Xyl-sinapoyl-Glc-Gal). Crude enzyme extracts prepared from these cells can catalyze the transfer of the sinapoyl group from sinapoylglucose to the 6-position of the glucose attached to the anthocyanin (Figure 2) [11,12]. Likewise, in *Chenopodium rubrum* cells in suspension cultures and in *Lampranthus sociorum* petals,* p*-coumaroylglucose and feruloylglucose are utilized as acyl-donors for betacyanins, another major class of plant pigment [13]. Identification of the genes for these acyl-glucose-dependent ATs was only achieved much later. In 2000, acyl-glucose-dependent ATs involved in diacyl-glucose biosynthesis in the wild tomato (*Lycopersicon pennellii*) and sinapoylmalate biosynthesis in *Arabidopsis thaliana* were characterized [14,15]. Analyses of their amino acid sequences revealed that these acyl-glucose dependent acyltransferases belong to the serine carboxypeptidase-like (SCPL) protein family. More interestingly, immunological and cell biological analyses showed the transition of this type of AT (termed here SCPL-AT) into the vacuole where they are expected to act to accumulate acyl-glucose(s) [16,17].

Initially, it was believed that the glycosylation reactions for production of anthocyanins were only catalyzed by UGTs, which can glycosylate a wide variety of plant secondary metabolites. However, another type of anthocyanin glucosyltransferase (GT) has been discovered [18]. Surprisingly, this new type of GT belongs to a separate protein family from UGTs, namely glycoside hydrolase family 1 (GH1), which characteristically hydrolyzes glycosides. This GH1 type glucosyltransferase (GH1-GT) transfers a glucosyl group to an anthocyanin using an acyl-glucose as the glucosyl donor. Glucosylation reactions mediated by a GH1-GT have now been identified in several species [17,19,20,21]. 

**Figure 3:**
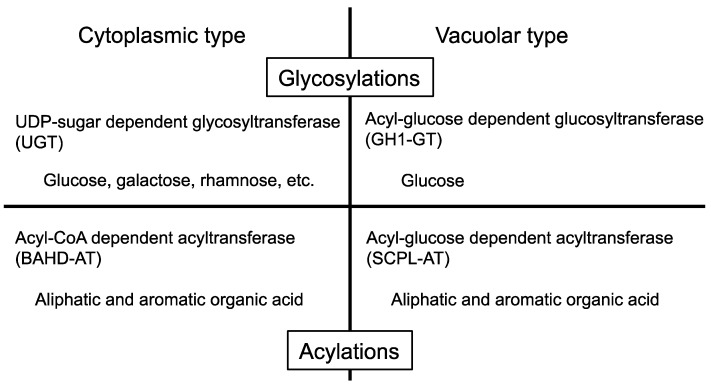
Classification of enzymes involved in anthocyanin modification.

Furthermore, the acyl-glucose dependent anthocyanin glucosyltransferase (AAGT) protein contains a signal peptide at its *N*-terminal end for translocation into the vacuole [17,18]. As a consequence of recent investigations, four types of enzyme have now been identified as mediating anthocyanin modification: two glycosyltransferases (GTs) and two acyltransferases (ATs), with one of each type functioning in the cytosol and the other in the vacuole [3] (Figure 3). Thus, the variable anthocyanin molecules in higher plants are created by the combined activities of these enzymes. Notably, both vacuolar enzymes,* i.e*., GH1-GT and SCPL-AT, make use of acyl-glucoses as the donor molecule [3].

As is outlined above, recent studies have clarified the contribution of acyl-glucoses to anthocyanin generation in higher plants. In the remainder of this review, we focus on the roles of acyl-glucoses as glucosyl and acyl donors in the anthocyanin biosynthetic pathways of carnation, delphinium and *Arabidopsis* in which the involvement of acyl-glucoses in both acylation and glucosylation of anthocyanin were shown.

## 2. UGTs Involved in Acyl-Glucose Generation

**Figure 4:**
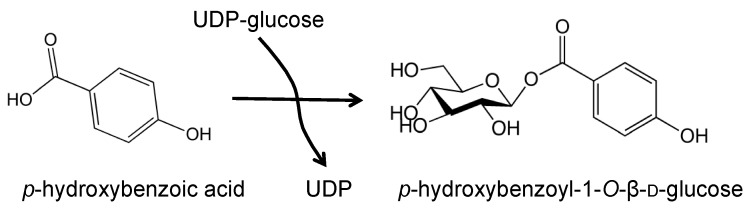
Representative UDP-glucose dependent glucosyltransferase (UGT) reaction for acyl-glucose synthesis.

As described above, acyl-glucose contributes to the diversity of secondary metabolites by acting as an energy-rich donor molecule in both transacylation and transglucosylation reactions. It is known that the biosynthesis of glucose esters is performed by UGTs (Figure 4). Acyl-glucoses mainly accumulate in the vacuole, although the glucosyltransferase reactions catalyzed by UGTs take place in the cytosol [22]. To date, a number of UGTs have been reported as being involved in the formation of acyl-glucose. In *Arabidopsis*, many of the UGTs necessary for synthesis of the different types of acyl-glucose have been identified. Lim* et al*. performed biochemical analyses of recombinant proteins* in vitro* and identified UGTs that exhibit GT activity to form an ester bond [23]. That study listed UGT84A1, UGT84A2 and UGT84A3 as showing significant activity to form glucose ester conjugates with hydroxycinnamic acids (HCAs), such as cinnamic acid, *p*-coumaric acid, caffeic acid, ferulic acid and sinapic acid. Although UGT84A1, UGT84A2 and UGT84A3 functionally overlap in their acceptor preferences* in vitro*, subsequent investigations have shown that UGT84A1 is involved in *p*-hydroxybenzoylglucose (pHBG) synthesis rather than HCA-glucose synthesis [24]. Analyses using overexpressing or functionally-deficient mutant lines show that UGT84A2 supplies sinapoylglucose, which acts as a bi-functional donor for anthocyanin modification [25], and that UGT84A3 seems to be involved in the synthesis of *p*-coumaroylglucose, which is associated with cell wall structure [26].

**Figure 5:**
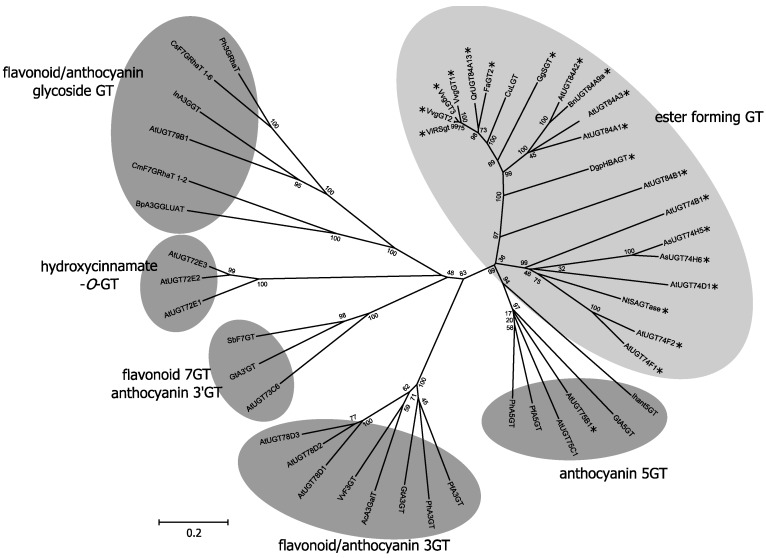
Phylogenetic tree analysis of UGTs mediating acyl-glucose production. The tree was constructed from multiple amino acid sequences using the Neighbor-Joining algorithm. The clade of the UGTs involved in acyl-glucose biosynthesis is shown with a gray circle and the others with dark gray circles. UGTs that exhibit acyl-glucose synthesis activity* in vitro* are marked with asterisks.

Phylogenetic analysis of the amino acids sequences of UGTs may provide some insights into their metabolic functions. An example of such an analysis of UGTs responsible for glucose ester and *O*-glucoside synthesis in higher plants is presented in Figure 5. This analysis shows that UGTs capable of glucose ester formation largely form a cluster (marked with an asterisk in Figure 5), although there are exceptions.

The accession numbers of the amino acid sequences are as follows: Ac3AGalT (AB103471), AtUGT72E1 (AL049862), AtUGT72E2 (AB018119), AtUGT72E3 (AF077407), AtUGT73C6 (NM_129234), AtUGT74B1 (BT001160), AtUGT74D1 (NP_180734), AtUGT74F1 (NP_973682), AtUGT74F2 (BT010327), AtUGT75B1 (NP_563742), AtUGT75C1 (AK226538), AtUGT78D1 (NM_102790), AtUGT78D2 (NM_121711), AtUGT78D3 (NM_121709), AtUGT79B1 (BT033073), AtUGT84A1 (BT015796), AtUGT84A2 (Q9LVF0), AtUGT84A3 (NP_193284), AtUGT84B1 (NM_127890), AsUGT74H5 (EU496509), AsUGT74H6 (EU496520), BnUGT84A9a (AF287143), BpA3GGLUAT (AB190262), CuLGT (Q9MB73), CmF7GRhaT 1-2 (AY048882), CsF7GRhaT 1-6 (DQ119035), DgpHBAGT (AB889521), FaGT2 (AY663785), GgSGT (AB362221), GtA3GT (D85186), GtA3'GT (AB076697), GtA5GT (AB363839), Ihant5GT (AB113664), In3GGT (AB192314), NtSAGTase (AF190634), PfA3GT (AB002818), PfA5GT (AB013596), PhA3GT (AB027454), PhA5GT (AB027455), Ph3GRhaT (X71059), QrUGT84A13 (KF527849), SbF7GT (AB031274), VlRSgt (ABH03018), VvF3GT (AF000371), VvgGT1 (JN164679), VvgGT2 (JN164680) and VvgGT3 (JN164681). Ac, *Aralia cordata*; As, *Avena strigosa*; At, *Arabidopsis thaliana*; Bn, *Brassica napus*; Bp, *Bellis perennis*; Cm, *Citrus maxima*; Cs, *Citrus sinensis*; Cu, *Citrus unshiu*; Dg, *Delphinium grandiflorum*; Fa, *Fragaria × ananassa*; Gg, *Gomphrena globosa*; Gt, *Gentiana triflora*; Ih, *Iris × hollandica*; In, *Ipomoea nil*; Nt, *Nicotiana tabacum*; Pf, *Perilla frutescens*; Ph, *Petunia × hybrida*; Qr, *Quercus robur*; Sb, *Scutellaria baicalensis*; Vl, *Vitis labrusca*; Vv, *Vitis vinifera*. Bar = 0.2 amino acid substitutions/site.

For example, UGT75B1 is located in a cluster composed of anthocyanin 5-*O*-glucosyltransferases; Lim* et al*. showed that UGT75B1 acts on the carboxyl group of hydroxybenzoic acids (HBAs), such as benzoic acid, 3-hydroxybenzoic acid, *p*-hydroxybenzoic acid (pHBA) and 3,4-dihydroxybenzoic acid [24]. UGT75B1 also catalyzes glucose ester formation in *p*-aminobenzoic acid (pABA), which contributes to the storage of pABA in the vacuole in the glucosylated form [27]. Although UGT74F1 can form a benzoate glucose ester* in vitro* [24], the* in vivo* role of this enzyme is suggested to be 2-*O*-glucosylation of salicylic acid [28]*.* Both VlRSgt of *Vitis labrusca* and CuLGT of *Citrus unshiu* have *O*-glucosyltransferase activity, although they are positioned in the ester-forming GT clade in the phylogenetic tree (Figure 5). It has been reported that VIRSgt also shows significant activity in the formation of glucose ester conjugates with both HCAs and HBAs [29,30]. Many UGTs seem to exhibit broad substrate specificities* in vitro* and to show enzymatic activity for substrates other than those in their* in vivo* metabolic pathway(s) [31,32,33]. Sometimes, such discrepancies lead to confusion in determining the* in vivo* roles of the enzymes. Thus, conclusions on the function of a UGT need to be based not only on* in vitro* enzymatic activity but also on the results from genetic, physiological and molecular biological analyses.

pHBG, the key compound for cyanodelphin and violdelphin biosynthesis in delphiniums, is supplied by a UGT, DgpHBAGT, that exhibits broad substrate specificity* in vitro* [34]. In DgpHBAGT depletion cultivars, delphinidin 3-*O*-rutinoside,* i.e*., 7-unmodified anthocyanin, is accumulated as the major anthocyanin because lack of pHBG prevents enzymes such as DgAA7GT generating anthocyanin 7-polyacylation [34]. Interestingly, these cultivars accumulate *p*-hydroxybenzoic acid 4-*O*-β-D-glucoside instead of pHBG (Figure 1). This may be the result of other UGT(s) showing broad substrate specificities and acting to glucosylate excess pHBA to detoxify and transport it into the vacuole. In wild type cultivars, the pHBA synthesized in the cytosol is efficiently glucosylated by DgpHBAGT for transport into the vacuole. Such flexibility in the metabolic roles of UGTs* in vivo* might be one mechanism for producing the diversity of secondary metabolites in plants. In addition to aromatic acyl-glucose, aliphatic acyl-glucose also acts as an active donor. Malylglucose is used as the donor of a malyl moiety in anthocyanin modification in carnations (described above). In addition to anthocyanin biosynthesis, isobutyrylglucose is known to be an energy-rich acyl-donor for glucose polyester generation by LpSCPL in the wild tomato (*L. pennellii*) [15,35]. Despite the importance of aliphatic acyl-glucoses, relatively little is known of the UGTs that are responsible for aliphatic acyl-glucose synthesis. FaGT2, which was first shown to be involved in cinnamoyl- and *p*-coumaroyl-glucose synthesis during the ripening of strawberry fruits, also exhibits the ability to glucosylate sorbic acid at very high rates and to glucosylate HCAs and xenobiotic compounds [36,37]. Future research on GTs involved in aliphatic acyl-glucose will undoubtedly provide new insights into the* in vivo* roles of aliphatic acylated compounds and their biosynthetic pathways in higher plants.

## 3. Acyl-Glucoses as Potential Intermediates for Secondary Metabolite Biosynthesis

Plant secondary metabolites commonly exist in their glycoside form in which aglycons bind to sugar group(s) through glycosidic (ether) linkages in the vacuole [38]. Some metabolites accumulate in the *C*-glycoside form [39], whereas others form compounds in which organic acid and glucose are bound to each other by ester bonding. Typical acyl-glucoses (acyl-1-*O*-β-D-glucose) are illustrated in Figure 2 (these substances are generally termed acyl-glucoses rather than glucosides). The major category of acyl-glucoses is that conjugating to HCAs and HBAs. For example, caffeoylglucose and coumaroylglucose accumulation occur in the fresh flowers of *Camellia reticulate* [40], while large amounts of sinapoylglucose and feruloylglucose respectively accumulate in carrot and silver beachtop cells in suspension cultures [11,12]. Likewise, accumulation of HCA-glucoses and HBA-glucoses has been reported in carnation petals [18]. All these acyl-glucoses are thought to be stored in the vacuole [41]. Plant species of the order Brassicaceae accumulate sinapoylglucose; additionally, acylated sinapoylglucose has also been detected in *Brassica napus* seeds [42]. Polyacylated acyl-glucose has been found in the leaves of *Rhus typhina* and dimeric *p*-coumaroylglucose has been extracted from *Petrorhagia velutina* [43,44]. Some studies have also reported other acyl-glucoses that are not derived from HCAs or HBAs. In the carnation, in addition to various aromatic acyl-glucoses, aliphatic acyl-glucose has also been found [45]. Aliphatic acyl-glucose also occurs in the glandular trichomes of the wild tomato (*Lycopersicon pennellii*), which secrete 2,3,4-tri-isobutyrylglucose to aid insect resistance [35].

It is clear from the above description that acyl-glucose molecules are widespread in the plant kingdom. These acyl-glucoses have a variety of functions, such as antifeedants, phytoanticipins and phytoalexins, signaling molecules, and UV protectants [46]. Thus, acyl-glucoses are physiologically important compounds in plants as they are the final, functional products of secondary metabolites. A possible role for acyl-glucoses has been proposed from the estimation of Gibb’s free energy changes that occur in acyl-glucose hydrolysis: acyl-glucoses may function as high-energy intermediates in the production of acylated secondary metabolites [47]. As described above, acyl-glucose dependent acyltransferase activities have been detected in several plants such as the carrot, silver beachtop, wild tomato, *C. rubrum*, and *L. sociorum.* One well-studied metabolic pathway involving acyl-glucose dependent acyltransferase is the biosynthesis of sinapoyl-derivatives in brassicaceous species such as *Arabidopsis*. Sinapoylmalate is a major sinapate ester in *Arabidopsis* that accumulates in the vacuoles of cells in the sub-epidermal tissue to provide shielding against the deleterious effects of UV-B irradiation [26]. Formation of the sinapoylmalate is mediated by sinapoylglucose:malate sinapoyltransferase (an SCPL-AT family protein). Sinapoylcholine, a major phenylpropanoid that accumulates in seeds and is utilized during germination, is generated by a similar enzyme, SCPL19, that transfers a sinapoyl group to choline from acyl-glucose [48]. SCPL19 can also transfer a benzoyl group to choline from benzoylglucose [49]. Arabidopsis seeds contain a range of related compounds, such as benzoylated glucosinolate and sinapoylated glucosinolate. A knockout mutant analysis showed that *SCPL17* was a candidate gene for benzoylated glucosinolate production. Sinapoylglucose dependent sinapoyltransferase can also synthesize the more complicated sinapoyl ester, 1,2-disinapoylglucose [46].

Recent investigations in our laboratory have identified another role for acyl-glucoses in anthocyanin modification. We found that several plant species glucosylate some anthocyanins in the vacuole with an enzyme that utilizes acyl-glucose as the glucosyl-donor [17,18,19,20,21]. That is, acyl-glucoses can be used not only as acyl-donors but also as glucosyl-donors. Our studies showed that pHBG can be used as a bi-functional donor for both acyl- and glucosyl-moieties for anthocyanin modification in delphinium [17]. The properties of the enzymes for this modification are described below.

## 4. Carnations

Carnations are the one of the most popular and big-market flower crops worldwide. Flower colors in carnations result from modification of the anthocyanin molecule, macrocyclicmalylanthocyanin (Figure 1b) [50,51,52,53]. The anthocyanin can be modified by two glucose molecules at the 3- and 5-positions and by formation of a bridge by a single molecule of malate between these moieties; this modified anthocyanin compound is thought to be limited to the family Caryophyllaceae [51].

**Figure 6:**
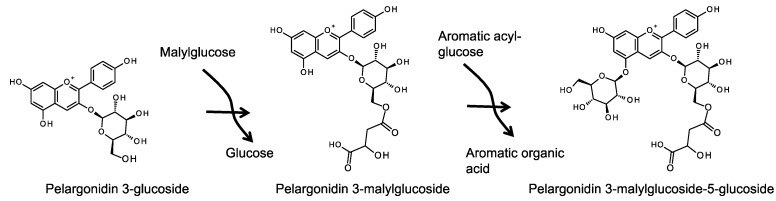
Anthocyanin malylation and glucosylation catalyzed by acyl-glucose dependent transferases in carnation.

Aliphatic acyl-group transfer reactions are generally catalyzed by a BAHD-AT enzyme. For example, bonding of a malonyl residue is mediated by an anthocyanin malonyltransferase, a type of enzyme that is now well characterized in several plant species [54]. The modification of anthocyanin with succinic acid, an aliphatic organic acid similar to malic acid (they differ in the hydroxyl group at the C-2 position), has been identified in the cornflower (*Centaurea cyanus*) [55,56,57]. The enzymatic activity of succinyl-CoA:anthocyanin 3-glucoside succinyltransferase has been characterized [58]; however, to date, the gene encoding this enzyme has not been identified. Although the information on the activity of this enzyme seems to support the view that the malyl transfer reaction is also catalyzed by an acyl-CoA dependent AT in carnations, a study in 2008 showed that the reaction was actually mediated by a distinctly different type of AT [45]. In a screen for the compound that acts as the malyl-donor for malyltransferase in carnation petals (Figure 6), Abe* et al*. identified malylglucose (not malyl-CoA) as the malyl-donor molecule in the anthocyanin malyl transfer reaction. 

A candidate gene for anthocyanin malyltransferase (AMalT) was identified in variegated carnation flowers using a transposon-tagging method [59]. AMalT is a member of the SCPL-AT family, which includes proteins that are known to act in the vacuole [16]. Interestingly, crude protein extracts prepared from carnation petals show AMalT activity toward anthocyanidin 3-glucosides, although only trace activity occurs when anthocyanin 3,5-diglucosides are used as the acyl acceptor. This suggests that 5-glucosylation may occur in the vacuole; this reaction is expected to be the next step after malylation catalyzed by a vacuolar enzyme.

The cDNA for the anthocyanin 3-glucosyltransferase that mediates the first modification step in macrocyclicmalylanthocyanin has been isolated from carnation petals [60]. Additionally, 18 UGT homologous cDNAs have also been isolated; however, the enzyme that mediates the 5-glucosylation step has not been identified, although anthocyanin 5-glucosyltransferases of other plant species have been identified [54]. Recently, Matsuba* et al*. reported that anthocyanin 5-glucosylation is performed by an enzyme that is distinctly different to UGT [18]. They showed that anthocyanin 5-glucosyltransferase activity could be detected in a crude enzyme extract prepared from carnation petals combined with an alcohol extract containing low molecular substances expected to include glucose donor substance(s). Vanillylglucose, a type of acyl-glucose, was identified as the glucosyl-donor molecule for the anthocyanin 5-glucosylation reaction in carnation petals (Figure 6). Subsequently, an enzyme showing acyl-glucose dependent anthocyanin 5GT (DcAA5GT) activity was purified and the amino acid sequence of its N-terminus was determined. The observation that this sequence lacks a first methionine implies that the protein contains a signal peptide at its N-terminus. Analysis of the deduced amino acid sequence corresponding to the full-length cDNA suggests that the enzyme belongs to the glycoside hydrolase family 1 (GH1); this family of proteins are known to mediate glycoside hydrolysis and to have a putative signal peptide at their N-terminal ends. In a transient expression experiment in onion epidermal cells, AA5GT fused to green fluorescence protein was found to localize to the vacuole [18].

The final steps of anthocyanin modification in carnations are likely to require the activities of both AMalT and DcAA5GT in the vacuole. AMalT is able to utilize anthocyanin 3-glusoside as the acceptor molecule but is less efficient at utilizing anthocyanin 3,5-diglucosides [45]. By contrast, DcAA5GT can recognize both anthocyanin 3-glucoside and 3-malylglucoside [18], suggesting that glucosylation of the 5-position occurs after malylation of the glucose moiety at the 3-position. However, it is still unclear why the AMalT reaction using anthocyanin 3-glucoside occurs prior to AA5GT reaction* in vivo*. Although both AMalT and DcAA5GT recognize anthocyanin 3-glucosides, the modification steps to generate macrocyclicmalylanthocyanin as the final product are catalyzed in the sequence of malylation and then glucosylation at the 5-position. If glucosylation of anthocyanin 3-glucoside occurs (an activity that has been observed* in vitro*) prior to malylation* in vivo*, then both macrocyclicmalylanthocyanin and anthocyanin 3,5-diglucosides would be expected to be synthesized and accumulated in petals. Petals of wild type carnations accumulate macrocyclicmalylanthocyanin but few 3,5-diglucosides could be detected; however, anthocyanin 3,5-diglucosides are synthesized and accumulated in AMalT defective mutants [59,61]. The order of this sequential reaction might result from the intrinsic properties of the enzymes or from the formation of weakly associated enzyme complex in the vacuole [62]. Future analyses of the proteins will undoubtedly provide further insights into this phenomenon.

## 5. Delphiniums

**Figure 7:**
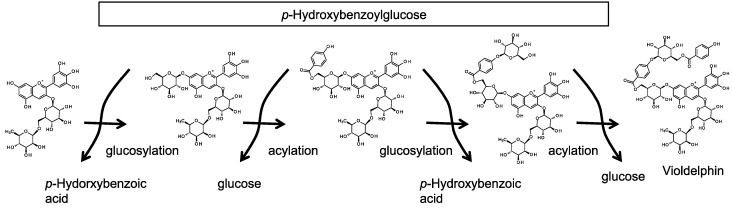
Concatenated units in violdelphin synthesized in a step-by-step reaction utilizing acyl-glucose as both glucosyl and acyl donor in delphinium.

Delphiniums are popular ornamental plants because of their characteristic blue flowers. Several mechanisms have been proposed to explain how anthocyanin molecules produce the bluish coloration of the sepals [2]. Delphinium sepals contain two major 7-polyacylated anthocyanins termed violdelphin and cyanodelphin [63,64]. The formation of face-to-face intramolecular stacking structures between anthocyanin chromophores and aromatic groups attached to the anthocyanins through their sugar groups can generate blue pigment complexes [2,64,65]. Violdelphin consists of a structure that contains two glucose moieties and pHBA groups at the 7-position (Figure 7). It is known that species such campanula (*Campanula medium*) and cineraria (*Senecio cruentus*), which have violet-blue flowers, accumulate delphinidin-based anthocyanins decorated with multiple aromatic acyl groups at the 7-position [2]. However, characterization of anthocyanin 7-glucosylation, the expected first step of modification at the 7-position, was not achieved until 2010. At that time, the anthocyanin 5-glucosylation mechanism was first clarified in carnations; subsequently, anthocyanin 7-glucosyltransferase was identified in delphinium by means of a homology based cloning method [18]. Anthocyanin 7-glucosyltransferase in delphinium (DgAA7GT) is a GH1-GT, as is DcAA5GT. AA7GTs have also recently been identified in agapanthus and campanula [20,21]. Agapanthus is a monocot, while delphinium and campanula belong to different dicot orders. The facts that AA7GTs are present in such evolutionarily diverse plant species and that anthocyanin 7-glucosylation mediated by UGTs has not been reported [54], supports the conclusion that anthocyanin 7-glucosylation is commonly mediated by GH1-GTs.

In delphinium, the modification steps after 7-glucosylation have been clarified in the biosynthesis of violdelphin [delphinidin 3-*O*-rutinoside-7-*O*-(6-*O*-(4-*O*-(6-*O*-(*p*-hydroxybenzoyl)-glucosyl)-oxybenzoyl)-glucoside)]. Analyses using a crude protein extract prepared from delphinium sepals showed that these modification steps are carried out in a step-by-step sequence in the order *p*-hydroxybenzoylation, a second glucosylation, and then a second *p*-hydroxybenzoylation [17]. All of the enzymatic reactions to generate violdelphin after the 7-glucosylation reaction are mediated by acyl-glucose dependent enzymes (SCPL-ATs and GH1-GT): *p*-hydroxybenzoylation is catalyzed by AA7G-AT; glucosylation is catalyzed by AA7BG-GT; and the second *p*-hydroxybenzoylation is catalyzed by AA7GBG-AT. The deduced amino acid sequences of these SCPL-ATs and GH1-GT contain a putative signal peptide for localization in the vacuole at their N-termini. This attribute of these enzymes was expected since it is reasonable to predict that the reactions that follow 7-glucosylation, which is mediated by a vacuolar type GH1-GT, should also occur in the vacuole. For the biosynthesis of violdelphin, all of the biosynthetic reactions are expected to occur sequentially and be dependent on pHBG (Figure 7). pHBG behaves as a bi-functional donor providing the glucosyl moiety for AA7GT and AA7BG-GT, and the acyl moiety for AA7G-AT and AA7GBG-AT; this bi-functionality has led to pHBG being referred to as a “Zwitter donor” in analogy to the concept of a Zwitterion [17].

Bluish delphinium sepals accumulate a remarkable amount of pHBG, but do not accumulate other aromatic acyl-glucoses, such as vanillyl-, isovanillyl-, caffeoyl-, *p*-coumaroyl-, sinapoyl- or feruloyl-glucose to a detectable level. Recombinant DgAA7GT and DgAA7BG-GTs (DgAA7BG-GT1 and DgAA7BG-GT2) proteins in *Escherichia coli* showed a donor preference for pHBG over other aromatic acyl-glucoses [17]. However, both AA7G-AT and AA7GBG-AT in crude extracts of delphinium sepals can use other HCA-glucose species as well as pHBG [17]. Despite this ability, there is no evidence that anthocyanins in delphiniums are modified using HCG molecules other than pHBG. The moieties attached to anthocyanins in delphiniums appear to be determined by the acyl-glucose species that accumulates in the vacuole rather than by the substrate preference of the AT. Thus, carrot cells and beach silvertop cells in suspension cultures accumulate Cya 3-(Xyl-sinapoyl-Glc-Gal) and Cya 3-(Xyl-feruloyl-Glc-Gal), respectively. Both anthocyanins are produced by acylation mediated by an acyl-glucose-dependent AT and, in both species, the ATs have a preference for feruloylglucose over sinapoyl-, caffeoyl- or *p*-coumaroyl-glucose* in vitro*, although they can acylate Cya 3-(Xyl-Glc-Gal) using different HCGs. Carrot cells mainly synthesize sinapoylglucose* in vivo,* while beach silvertop cells synthesize feruloylglucose; these accumulated acyl-glucoses are used as acyl-donors to modify Cya 3-(Xyl-Glc-Gal) resulting in the determination of the major acylated anthocyanin molecule* in vivo* [12]. Furthermore, recombinant delphinium AA7BG-GTs do not recognize artificially synthesized delphinidin 3-*O*-rutinoside-7-*O*-(6-*O*-(*p*-coumaroyl)-glucoside) as an acceptor substance *in vitro* [17]. The evolution of the ability of GT and AT to use pHBG as a common donor substance for glucosylation and acylation may underlie the generation of complicated anthocyanin species like violdelphin and cyanodelphin in delphiniums.

## 6. *Arabidopsis*

The leaves and stems of *Arabidopsis* accumulate anthocyanins that have been modified with multiple sugar and organic acid moieties when they are cultivated under stressful conditions. The exact function(s) of anthocyanin induced by biotic and abiotic stress have still remained controversial, while the contribution to protect against several stresses such as light, UV, and free radicals are proposed [66]. The major anthocyanin structure is cyanidin 3-*O*-(2''-*O*-(2'''-sinapoyl)-xylosyl)-6''-*O*-(*p*-*O*-(glucosyl)-*p*-coumaroyl)-glucoside) 5-*O*-(6''''-*O*-(malonyl) glucoside), generally termed A11 (Figure 1c) [67]; the ten precursor structural anthocyanins of A11 have also been determined [68]. Three UGTs, one AAGT, two BAHD-ATs and one SCPL-AT are required for A11 construction. The genes encoding these anthocyanin modification enzymes have also been identified [19].

**Figure 8:**
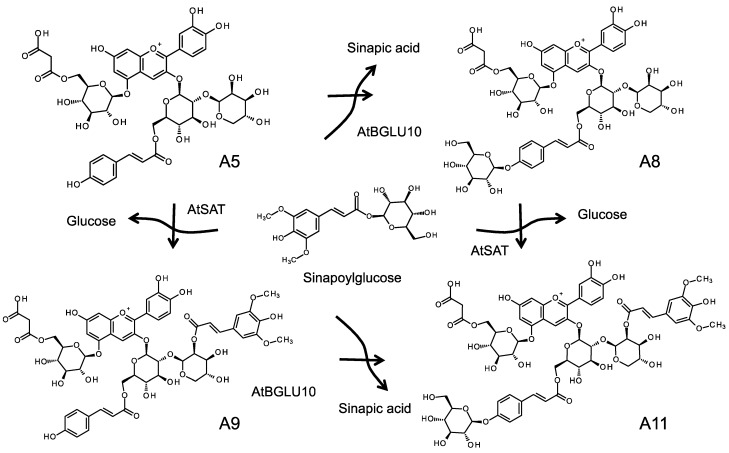
Sinapoylation and the glucosylation of the *p*-coumaroyl moiety on the anthocyanin A11 is catalyzed by enzymes using sinapoylglucose as a Zwitter donor in *Arabidopsis*.

Anthocyanin modification in *Arabidopsis* commences with the glucosylation of the 3-position of the anthocyanin aglycon, cyanidin. UGT78D2 (At5g17050) catalyzes 3-glucosylation of both flavonol and anthocyanidin in the cytosol [69,70]. After this glucosylation step, two possible reactions may occur. Two anthocyanin ATs (At3AT1: At1g03940, At3AT2: At1g03495) that mediate *p*-coumaroylation of the C6 position of the glucosyl group on anthocyanin have been identified. These At3ATs are BAHD-AT type enzymes that use acyl-CoA as the acyl-donor and have a preference for cyanidin 3-glucoside over cyanidin 3,5-diglucoside [71]. Xylosylation at the 2''-position of cyanidin 3-glucoside is catalyzed by UGT79B1 (At5g54060). UGT79B1 also recognizes cyanidin 3-glucoside as an acceptor substrate but not cyanidin 3,5-diglucoside [25]. In an *Arabidopsis* mutant deficient for the *UGT79B1* gene, anthocyanin A11 lacking the sinapoylxylose moiety accumulated. This suggests the possibility of two reaction routes: xylosylation occurs first; or, *p*-coumaroylation occurs in advance of xylosylation. However, the modification steps at the 5-position are believed to occur after these reactions because At3AT1, At3AT2 and UGT79B1 are all able to use cyanidin 3-glucoside as an acceptor substance but not cyanidin 3,5-diglucoside [25,71]. The 5-position of anthocyanidin is glucosylated by UGT75C1 (At4g14090) and then malonylated by malonyl-CoA: anthocyanidin 5-*O*-glucoside-6''-*O*-malonyltransferase (At5MAT: At3g29590) [68,72]. A *UGT75C1* knockout mutant was found to accumulate deglucosylated anthocyanins at the 5-position but its 3-position was modified similarly to A11 [72]. An *At5MAT* knockout mutant was found to accumulate de-malonylated A11. These results suggest that the modification at the 5-position occurs irrespective of the 3-position modification. Since these modification steps are mediated by enzymes that are active in the cytoplasm and that use UDP-sugars as UGTs or acyl-CoA for BAHD-ATs (Figure 3), then construction of cyanidin 3-(2''-*O*-(xylosyl)-6''-*O*-(*p*-coumaroyl)-glucoside) 5-(6'''-*O*-(malonyl) glucoside), termed A5, is also likely to be carried out in the cytoplasm (Figure 8). A5 is then subjected to further modification reactions by vacuolar type enzymes.

Sinapoylation of the xylosyl group is catalyzed by sinapoylglucose:anthocyanin sinapoyltransferase (AtSAT: At2g23000), an SCPL-AT type of protein. Analysis of an *AtSAT* knockout mutant showed the accumulation of the de-sinapoylated anthocyanins A5 and A8 (glucosylated A5) (Figure 8) [46]. Until recently, the glucosylation mechanism of the hydroxyl group on *p*-coumaroyl residue was unclear. However, *AtBGLU10* (At4g27830) has now been identified as the gene encoding the enzyme for this glucosylation [19]. AtBGLU10 had been identified as a β-glucosidase with an unknown function. Acyl-glucose-dependent GT activity toward A9 has been found in a crude protein extract prepared from wild type *Arabidopsis* leaves that had been induced to synthesize anthocyanin under high light intensity conditions. In this reaction, sinapoylglucose was also used as the glucosyl donor for glucosylation at the *p*-coumaroyl group in the* in vitro* enzyme reaction. No activity is detected in protein extracts prepared from similarly treated leaves of *AtBGLU10* knockout mutant plants that mainly accumulate A9 (sinapoylated at the xylosyl group of A5; Figure 8) [19]. On the basis of these results, we conclude that AtBGLU10 mediates glucosylation of the *p*-coumaroyl group of A9. In addition, the fact that *AtSAT* knockout mutant line accumulates both A5 and A8 anthocyanins indicates that AtBGLU10 can recognize both A5 and A9* in vivo* [46]. Hence, analysis of anthocyanin structures in *Arabidopsis* knockout mutants indicates that vacuolar type enzymes might have a broad acceptor recognition that leads to the formation of a metabolic grid, unlike the situation in carnations in which there is a strict sequential order of acylation followed by glucosylation at the 5-position (Figure 8) [25]. Evidence from reverse genetic studies and biochemical analyses indicates these anthocyanin modification enzymes may be able to complete A11 through multiple pathways, e.g., it is possible that 5-modification occurs after the 3-modification. However, considering the sub-cellular localization of the enzymes involved in the processes, it seems reasonable to suggest that A5 is first generated in the cytoplasm and that sinapoylation and glucosylation subsequently occur in the vacuole after anthocyanin transportation. Both vacuolar type anthocyanin modification enzymes utilize acyl-glucose as the donor molecule. The exclusive accumulation of sinapoylglucose in *Arabidopsis* suggests that this compound is a unique donor molecule unlike pHBG in delphinium which is a Zwitter donor [17,19,26,46].

## 7. Perspectives and Conclusions

This review has mainly focused on the roles of acyl-glucoses as substrates for anthocyanin biosynthesis. Although the vacuole was initially regarded simply as a storage organ that accumulated a wide range of substances, recent studies show that it is also the location of diverse enzymatic reactions. The modification reactions mediated by vacuolar enzymes are not limited to anthocyanins [13,14,15,16,35,41,42,43]. Vacuolar enzymes are also involved in acylation reactions that utilize acyl-glucoses for the biosynthesis of other compounds [73]. With regard to acyl-glucose-dependent GTs, the acceptor molecules are not limited to anthocyanins. A GH1 protein that has the ability to catalyze the attachment of a glucosyl group from an acyl-glucose to phenylpropanoids, flavonoids, and phytohormones has been identified, suggesting that GH1-GTs might be required for glucosylation of compounds other than anthocyanins [74]. However, GH1-GTs that transfer sugar moieties other than glucose and that utilize other acylated sugars, such as acyl-xylose and acyl-galactose, have not yet been identified. The question remains to be answered whether acyl-sugars (other than acyl-glucose), for example, acyl-galactose and acyl-xylose, can be used by other GH1-related GTs as sugar donors and what roles these accumulated acyl-sugars play in the metabolism of higher plants. Another important question requiring further attention is the evolution of GH1-GTs in plants. Possibly, clarification of the mechanism by which glucoside hydrolase activity is changed to glucosyl transfer activity will lead to a more complete understanding of the origin of these enzymes.

Recent studies indicate the dynamic function of the vacuole as an active site of synthesis of plant metabolites. In future work, it will be important to verify that these enzymatic reactions truly occur in the vacuole as opposed to other organelles such as the pre-vacuole [38]. The question of how the reactions are mediated efficiently in the correct order also needs to be clarified. For example, cyanodelphin in delphinium requires nine synthetic steps mediated by vacuolar proteins [17]. Possibly, the enzymes involved in this biosynthetic process in the vacuole are associated to enable sequential and efficient metabolic reactions, as has been reported for other pathways in the cytoplasm. Further biochemical and cell biological analysis of vacuolar enzymes will contribute to our understanding of many aspects of enzymatic reactions in this organelle.
